# Diagnostic and prognostic utility of soluble CD 14 subtype (presepsin) for severe sepsis and septic shock during the first week of intensive care treatment

**DOI:** 10.1186/s13054-014-0507-z

**Published:** 2014-09-05

**Authors:** Michael Behnes, Thomas Bertsch, Dominic Lepiorz, Siegfried Lang, Frederik Trinkmann, Martina Brueckmann, Martin Borggrefe, Ursula Hoffmann

**Affiliations:** First Department of Medicine, University Medical Centre Mannheim (UMM), Faculty of Medicine Mannheim, University of Heidelberg, Theodor-Kutzer-Ufer 1-3, 68167 Mannheim, Germany; Institute of Clinical Chemistry, Laboratory Medicine and Transfusion Medicine, Paracelsus Medical University, Nuremberg, Germany; Boehringer Ingelheim GmbH & Co. KG, Ingelheim am Rhein, Germany

**Keywords:** ■■■

## Abstract

**Introduction:**

The aim of this study was to evaluate the diagnostic and prognostic value of presepsin in patients with severe sepsis and septic shock during the first week of ICU treatment.

**Methods:**

In total, 116 patients with suspected severe sepsis or septic shock were included during the first 24 hours of ICU treatment. Blood samples for biomarker measurements of presepsin, procalcitonin (PCT), interleukin 6 (IL-6), C reactive protein (CRP) and white blood cells (WBC) were drawn at days 1, 3 and 8. All patients were followed up for six months. Biomarkers were tested for diagnosis of sepsis, severe sepsis, septic shock and for prognosis of 30-days and 6-months all-cause mortality at days 1, 3 and 8. Diagnostic and prognostic utilities were tested by determining diagnostic cutoff levels, goodness criteria, C-statistics and multivariable Cox regression models.

**Results:**

Presepsin increased significantly from the lowest to most severe sepsis groups at days 1, 3 and 8 (test for linear trend *P* <0.03). Presepsin levels revealed valuable diagnostic capacity to diagnose severe sepsis and septic shock at days 1, 3 and 8 (range of diagnostic area under the curves (AUC) 0.72 to 0.84, *P* = 0.0001) compared to IL-6, PCT, CRP and WBC. Goodness criteria for diagnosis of sepsis severity were analyzed (≥sepsis, cutoff = 530 pg/ml; ≥severe sepsis, cutoff = 600 pg/ml; ≥septic shock, cutoff = 700 pg/ml; *P* <0.03). Presepsin levels revealed significant prognostic value for 30 days and 6 months all-cause mortality (presepsin: range of AUC 0.64 to 0.71, *P* <0.02). Patients with presepsin levels of the 4^th^ quartile were 5 to 7 times more likely to die after six months than patients with lower levels. The prognostic value for all-cause mortality of presepsin was comparable to that of IL-6 and better than that of PCT, CRP or WBC.

**Conclusions:**

In patients with suspected severe sepsis and septic shock, precipices reveals valuable diagnostic capacity to differentiate sepsis severity compared to PCT, IL-6, CRP, WBC. Additionally, presepsin and IL-6 reveal prognostic value with respect to 30 days and 6 months all-cause mortality throughout the first week of ICU treatment.

**Trial registration:**

ClinicalTrials.gov NCT01535534. Registered 14 February 2012.

## Introduction

Severe sepsis and septic shock represent major challenges of modern intensive care medicine, and still recently published international guidelines demand ongoing research about the pathophysiology, diagnostics and treatment [1]. Worldwide at least 19 million people are estimated to suffer from severe sepsis [2] and - if ever - only half of these patients are treated according to best standard of care even in the Western world [3]. Today, alert and earliest timing of diagnosis and treatment is still recommended as the best method of choice to prevent severe sepsis and septic shock. No single new effective medical therapy or decisive diagnostic tool has been found over the last decades [3]. Additionally, the increasing number of patients surviving severe sepsis or septic shock is endangered by an adverse long-term prognosis and therefore these patients need to be increasingly focused upon [1,3]. A broad range of clinical and laboratory parameters are specifically combined and define the diagnostic standard of severe sepsis and septic shock [1]. However, there is a great lack of evidence for biomarkers to reliably diagnose and predict the future course of patients suffering from severe sepsis or septic shock [4-6].

Soluble cluster of differentiation 14 subtype (sCD14-ST) - so-called presepsin - is cleaved from the monocyte/macrophage-specific CD14 receptor complex after binding with lipopolysaccharides (LPS) and LPS binding protein (LPB) during systemic infections [7-10]. Presepsin appears to reveal significant diagnostic capacity to diagnose sepsis, severe sepsis and septic shock compared to procalcitonin (PCT) in patients presenting to the emergency department [11-14]. However, a comparative evaluation of the diagnostic and prognostic implications between presepsin and PCT, interleukin 6 (IL-6), white blood cells (WBC) as well as C-reactive protein (CRP) in patients with severe sepsis and septic shock being treated on an internal ICU has rarely been investigated within one single study. Therefore, this study aims to comparatively evaluate the diagnostic, as well as short- and long-term prognostic utility of presepsin in patients with severe sepsis and septic shock during the first week of intensive care treatment.

## Materials and methods

### Study patients, design and data collection

The Mannheim Sepsis Study (MaSep, clinicaltrials.gov identifier: NCT01535534) was conducted as a mono-centric prospective controlled study at the University Medical Centre Mannheim (UMM), Germany. Patient enrolment started in October 2011. The study was carried out according to the principles of the declaration of Helsinki and was approved by the medical ethics commission II of the Faculty of Medicine Mannheim, University of Heidelberg, Germany. Informed consent was obtained from all participating patients or their legal representatives.

The study was designed to reflect a representative cohort of patients with a minimum age of 18 years, who had proven criteria of severe sepsis or septic shock, found at a typical internal ICU. Main exclusion criteria were any traumatic or postoperative cause of sepsis development (that is, poly-trauma, cerebral trauma, critical postoperative status, or burns). Diagnosis of systemic inflammatory response syndrome (SIRS) and of sepsis severity was based on established criteria [15,16]: when patients revealed a microbiologically or clinically proven infection, they were assigned to the sepsis group. Patients were categorized to the severe sepsis group if they developed at least one of the following newly developed, sepsis-induced organ failures: acute encephalopathy, pulmonary organ failure defined as the ratio of the partial pressure of oxygen (PaO_2_) to the fraction of inspired oxygen (FiO_2_) PaO_2_/FiO_2_ < 250, renal organ failure with urine output <0.5 ml/kg/h, hematological organ failure with platelet count <100,000/mm^3^ or unexplained metabolic acidosis with pH <7.3 and lactate levels >1.5 times the upper limit of normal. Sepsis-induced organ failures in these patients were strongly connected to infection and were present for less than 24 h. Patients developing cardiovascular organ failure with need for vasopressors longer than 1 h were categorized as suffering from septic shock. Disease severity on the ICU was documented by the acute physiology and chronic health evaluation II (APACHE II) and the sequential organ failure assessment (SOFA) score [17,18].

All patient data, such as creatinine levels, hemoglobin, hematocrit, WBC count, platelet count, CRP, bilirubin, sodium, potassium, urea, IL-6, PCT, body temperature, respiratory rate, heart rate, blood pressure, partial pressure of O2 and CO2, bicarbonate, base excess, lactate, pH value, Glasgow coma scale (GCS) were documented. Additionally, prior medical history, age, sex, body weight and the germ spectrum were documented.

After the end of each hospital treatment, two study physicians independently reviewed all available clinical data of the study patients and classified all patients into four disease groups: SIRS, sepsis, severe sepsis or septic shock. The study physicians were blinded to the results of tested biomarker measurements, such as presepsin, PCT and IL-6.

Blood samples for presepsin measurements were taken within 24 h after clinical onset of severe sepsis or septic shock on the ICU (day 1) as well as on day 3 and 8 of ICU treatment. All patients were followed up until 30 days and 6 months after study inclusion by direct telephone visits with the patients or their general practitioners. The main prognostic outcome was all-cause mortality after 30 days and 6 months: 60 people without any clinically proven systemic infection served as a control group.

### Biomarker measurements

Blood samples were obtained by venipuncture into serum and ethylenediaminetetraacetic acid (EDTA) monovettes® (SARSTEDT AG & Co.; Nümbrecht, Germany). Within 30 minutes all blood samples were centrifuged at 1,000 × *g* at 4°C for 15 minutes. Serum/plasma was separated, frozen and stored at −80°C.

Presepsin measurements were performed with the PATHFAST® immunoassay analytical system (PROGEN Biotechnik GmbH, Germany; Mitsubishi Chemical Medience Corporation, Japan) using plasma from EDTA monovettes® [8,19]. IL-6 and PCT were measured in serum. IL-6 was measured with reagents from Roche Diagnostics (Roche Diagnostics, Mannheim, Germany) and PCT was measured with reagents from Thermo Fisher Scientific (Thermo Fisher Scientific Clinical Diagnostics, BRAHMS GmbH, Henningsdorf, Germany). The assays were performed on a Cobas e601 twin module (Roche Diagnostics, Mannheim, Germany). IL-6 and PCT measurements were performed at the central laboratory in Nuremberg, Germany.

### Statistical analysis

For normally distributed data, the Student *t*-test was applied. Otherwise, the Mann–Whitney *U*-test was used as a nonparametric test. Deviations from a Gaussian distribution were tested by the Kolmogorov-Smirnov test. Spearman’s rank correlation for nonparametric data was used to test the association of presepsin blood levels with medical parameters. Qualitative parameters were analyzed by use of a 2 × 2 contingency table and Chi^2^ test or Fisher’s exact test as appropriate. Quantitative data are presented as mean ± standard error of mean (SEM) or as median and interquartile ranges (25th to 75th percentiles), depending on the distribution of the data. For qualitative parameters absolute and relative frequencies are presented. A test for linear trend was applied to compare the biomarker levels in the different groups of sepsis severity. Post-hoc statistical power analyses were performed. All analyses were exploratory and utilized a *P*-value of 0.05 (two-tailed) for significance.

### Diagnostic value of biomarkers

For *C*-statistics: receiver-operating characteristic (ROC) curve analyses were performed with calculation of area under the curve (AUC) for diagnosis of sepsis, severe sepsis and septic shock during the first week of ICU treatment at days 1, 3 and 8. A minimal AUC was set at 0.75 to define valuable discriminative diagnostic capacity of any biomarker. Accordingly, diagnostic goodness criteria (that is, accuracy, specificity, sensitivity, negative/positive predictive values (NPV/PPV), and relative risk) of the biomarkers were calculated. Accuracy was defined as the sum of true positives plus true negatives divided by all measured patients. Diagnostic AUCs were compared by the method of Hanley *et al*. [20].

### Prognostic value of biomarkers

For *C*-statistics: ROC analysis with calculation of the AUC was performed for prognosis of all-cause mortality in all patients after 30 days and 6 months for all biomarkers (that is presepsin, IL-6, PCT, CRP, WBC), APACHE II and SOFA score. Prognostic AUCs were compared by the method of Hanley *et al*. [20]. Log-transformed biomarker concentrations over time (days 1, 3, 8) in survivors and non-survivors were analyzed by two-way analysis of variance (ANOVA) to estimate the effects of the two factors, time and survival, on biomarker levels. Kaplan-Meier survival curves according to presepsin quartiles were created and the corresponding hazard ratios (HR) were calculated for each quartile. Cox regression analysis was performed to adjust the prognostic value of presepsin and IL-6 with age, sex, creatinine, APACHE II and SOFA score and the number of ICU treatment days, all representing confounding factors on presepsin levels and objective clinical factors limiting prognosis of patients with severe sepsis and septic shock. HR are indicated for a log unit-change of presepsin or IL-6.

The calculations were performed with InStat and StatMate (GraphPad Software), SPSS software (SPSS Software GmbH), and SAS version, release 9.2 (SAS Institute Inc. Cary, NC, USA).

## Results

Baseline characteristics are given in Table 1. A total of 116 patients have been included into the MaSep study: 24% of the patients (n = 28) suffered from severe sepsis and 64% of the patients (n = 74) suffered from septic shock at the time of study entry. There were 12% of the patients classified to the sepsis (n = 5) or SIRS group (n = 9). In SIRS patients either no evidence of any infection was finally found or organ dysfunction was mainly caused by concomitant, manifested chronic pulmonary or acute neurological diseases.

**Table 1:** **Baseline characteristics of the Mannheim Sepsis Study (MaSep)**

	**Controls**	**SIRS**	**Sepsis**	**Severe sepsis**	**Septic shock**
	**(n = 60)**	**(n = 9)**	**(n = 5)**	**(n = 28)**	**(n = 74)**
**Age,** years (mean, range)	62 (42 to 87)	74 (61 to 81)	66 (50 to 81)	66 (26 to 87)	68 (26 to 88)
**Gender,** n (%)					
Male	29 (48)	5 (56)	4 (80)	21 (75)	52 (70)
Female	31 (52)	4 (44)	1 (20)	7 (25)	22 (30)
**Site of infection,** n (%)					
Lung	-	-	5 (100)	20 (71)	41 (55)
Urinary tract	-	-	-	3 (11)	4 (5)
Abdominal	-	-	-	3 (11)	12 (16)
Central nervous system	-	-	-	-	-
Skin	-	-	-	1 (4)	3 (4)
Heart	-	-	-	-	-
Neutropenia	-	-	-	-	-
Blood	-	-	-	1 (4)	7 (10)
Others	-	-	-	-	7 (10)
**Laboratory values,** mean ± SEM					
White blood cells, 10^9^/L	-	14.5 ± 1.7	19.2 ± 3.1	17.4 ± 3.1	19.5 ± 1.8
Platelets, 10^9^/L	-	210 ± 216	305 ± 202	218 ± 214	191 ± 142
Bilirubin, mg/dl	-	0.8 ± 0.2	0.5 ± 0.1	1.1 ± 0.3	2.9 ± 0.7
Creatinine, mg/dl	-	1.1 ± 0.1	1.2 ± 0.2	2.4 ± 0.3	2.7 ± 0.2
C-reactive protein, mg/L	-	68 ± 16	155 ± 28	178 ± 24	197 ± 12
Procalcitonin, ng/ml	-	2.0 ± 0.9	4.3 ± 2.8	6.9 ± 2.0	22.2 ± 4
Interleukin 6, pg/ml	-	335 ± 154	142 ± 53	1,385 ± 829	21,089 ± 15,437
pCO2, mmHg	-	43 ± 5	49 ± 14	45 ± 4	44 ± 2
Positive blood cultures, n (%)	-	0 (0)	0 (0)	8 (29)	25 (34)
**ICU parameters,** mean ± SEM					
ICU days	-	10 ± 2	8 ± 2	10 ± 2	15 ± 2
Ventilation days	-	3 ± 1	4 ± 2	6 ± 2	9 ± 2
Catecholamine days	-	2 ± 1	0 ± 0	2 ± 1	7 ± 1
Renal replacement therapy days	-	0 ± 0	0 ± 0	1 ± 0.6	3 ± 1
**APACHE II,** mean ± SEM	-	24 ± 2	18 ± 3	20 ± 2	27 ± 1
**SOFA score,** mean ± SEM	-	7.6 ± 1.1	6.2 ± 1.8	6.1 ± 0.5	11.8 ± 0.4
**All-cause mortality,** n (%)					
**30 days**					
Death	0 (0)	4 (44)	2 (40)	10 (36)	42 (57)
Survivor	60 (100)	5 (56)	3 (60)	18 (64)	32 (43)
**6 months**					
Death	0 (0)	4 (44)	3 (60)	12 (43)	53 (72)
Survivor	60 (100)	5 (56)	2 (40)	16 (57)	21 (28)

SIRS, systemic inflammatory response syndrome; SEM, standard error of the mean; APACHE II, acute physiology and chronic health evaluation II; SOFA, sequential organ failure assessment.

Mean APACHE II score at day 1 was highest in patients with septic shock (mean ± SEM = 27 ± 1). The most common primary site of infection was the lung in at least 50% of patients in each group, followed by abdominal and urinary tract infections (up to 16% of patients).

### Associations of presepsin with clinical and laboratory parameters

Presepsin levels were significantly correlated with clinical and laboratory parameters at day 1. As shown in Table 2, presepsin correlated with creatinine levels (*r* = 0.28, *P* = 0.002) as well as with the days of renal replacement therapy (RRT) during ICU treatment (*r* = 0.36, *P* = 0.0001). Additionally, presepsin correlated with WBC, CRP, PCT, IL-6 and bilirubin. Interestingly, presepsin was also significantly correlated with the number of days of intensive care treatment, mechanical ventilation and catecholamine therapy (*P* <0.05). Presepsin levels were not correlated with patients’ age and gender in this cohort (*P* >0.05) (data not shown).

**Table 2:** **Univariate correlations of presepsin with laboratory and clinical parameters in all patients (n = 116) at day 1**

	**r**	***P*** **-value**
**Creatinine**	0.28	0.002
**Bilirubin**	0.20	0.04
**White blood cells (WBC)**	0.17	0.07
**Platelets**	0.09	0.4
**C reactive proteine (CRP)**	0.22	0.02
**Procalcitonin (PCT)**	0.36	0.0001
**Interleukin 6 (IL-6)**	0.39	0.0001
**pCO2**	−0.25	0.007
**Systolic blood pressure**	−0.19	0.04
**Intensive care days**	0.22	0.02
**Mechanical ventilation days**	0.19	0.04
**Renal replacement days**	0.36	0.0001
**Catecholamines days**	0.21	0.03
**SOFA score**	0.23	0.02
**APACHE II score**	0.28	0.004

APACHE II, acute physiology and chronic health evaluation II; SOFA, sequential organ failure assessment.

### Diagnostic value of presepsin

**Figure 1:**
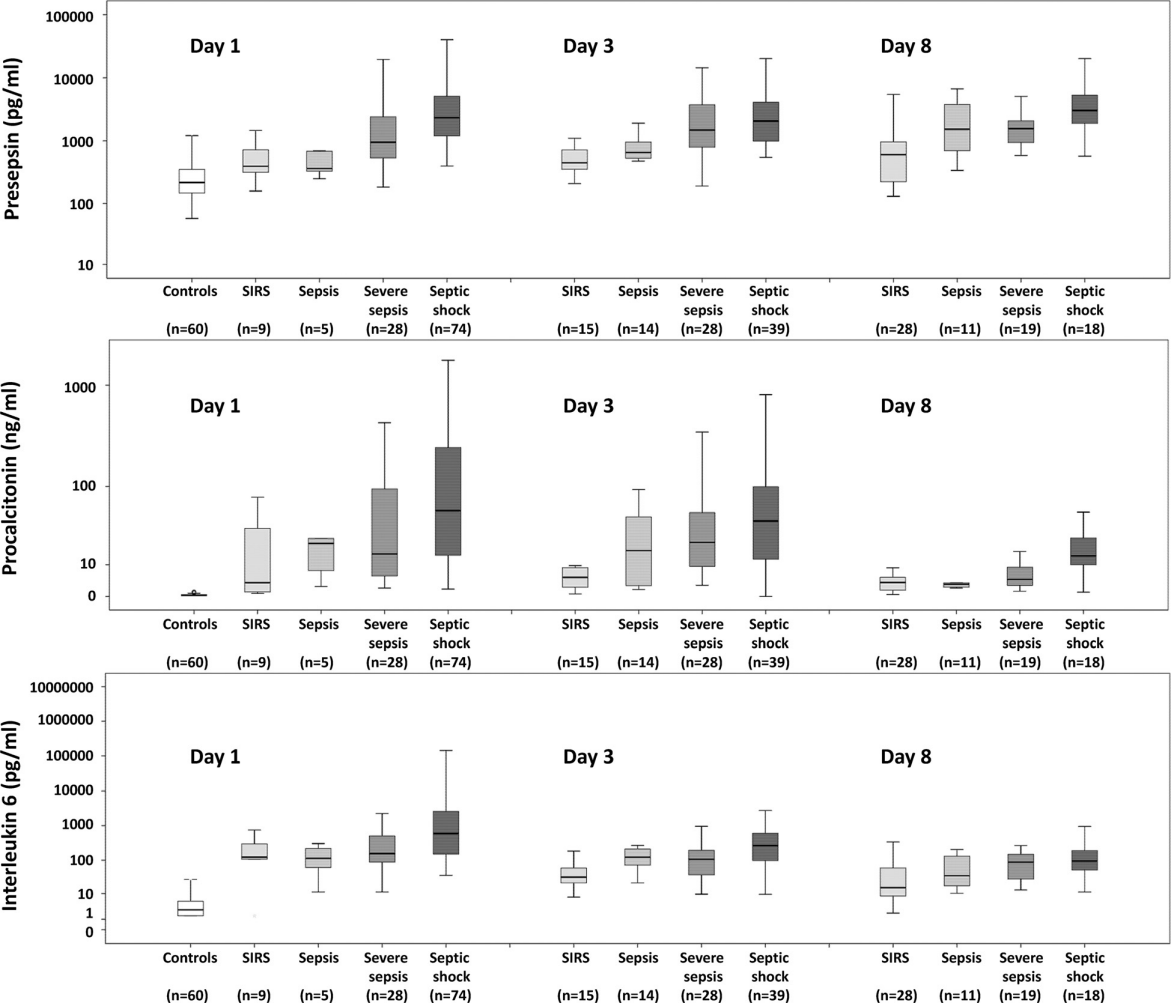
**Presepsin (top), procalcitonin (PCT, middle) and IL-6 plasma levels (bottom) in patients admitted to the internal ICU with proven criteria of systemic inflammatory response syndrome (SIRS), sepsis, severe sepsis and septic shock.** Left diagrams show results of biomarker measurements at day 1, middle diagrams show results at day 3 and right diagrams show results at day 8. Sixty individuals served as a control group at day 1. Data are presented as medians with 25th and 75th percentiles (boxes) and 5th and 95th percentiles (whiskers).

Figure 1 illustrates distribution of presepsin, IL-6 and PCT levels according to the different groups of sepsis severity at days 1, 3 and 8. A significant increasing trend of presepsin levels was observed compared to controls in the lowest to highest groups of sepsis severity during the first week of intensive care treatment (*P* ≤0.03), which was not observed for PCT or IL-6 (*P* >0.05).

Presepsin levels (pg/ml) were as follows (that is, median (IQR)): day 1: SIRS 393 (249 to 745), sepsis 362 (249 to 745), severe sepsis 947 (523 to 2,486), septic shock 2,330 (1,181 to 5,219); day 3: SIRS 448 (350 to 844), sepsis 651 (523 to 1,430), severe sepsis 1,479 (787 to 3,811), septic shock 2,060 (954 to 4,114); day 8: SIRS 604 (223 to 965), sepsis 1,528 (573 to 4,539), severe sepsis 1,556 (859 to 2,462), septic shock 3,041 (1,757 to 5,407); and controls 216 (146 to 350).

The diagnostic value of presepsin levels to diagnose septic shock (AUC = 0.80) was comparable to that of IL-6 (AUC = 0.86) and PCT (AUC = 0.83) at day 1 of ICU treatment (AUC differences, *P* >0.05) (Table 3). At day 3 of ICU treatment, the diagnostic value of presepsin (AUC = 0.84) to diagnose at least sepsis was significantly better than that of PCT (AUC = 0.69) (AUC difference, *P* = 0.05) and comparable to that of IL-6 (AUC = 0.81) (AUC difference, *P* >0.05). Interestingly, presepsin (AUC = 0.80) levels still revealed valuable diagnostic capacity to diagnose at least severe sepsis when compared to IL-6 (AUC = 0.71) and PCT (AUC = 0.66) at day 3. However, presepsin was not able to differentiate septic shock at day 3 (AUC = 0.72; that is, <0.75 predefined AUC margin), whereas the AUC of IL-6 was 0.76 at day 3. At day 8 of ICU treatment, the diagnostic value of presepsin was evident for all different groups of sepsis severity (for example, diagnosis of at least sepsis, presepsin AUC = 0.82), whereas IL-6, PCT, WBC and CRP did not exceed an AUC ≥0.75 (Table 3).

**Table 3:** **Diagnostic performance of biomarkers for diagnosis of severe sepsis and septic shock at days 1, 3 and 8 of ICU treatment, analyzed as area under the curve (95% CI)**

	**Presepsin**	**Interleukin-6**	**Procalcitonin**	**C-reactive protein**	**White blood cells**
**Day 1**					
Septic shock (n = 74)	**0.80 (0.73, 0.86)**	**0.86 (0.80, 0.91)**	**0.83 (0.77, 0.90)**	0.62 (0.49, 0.74)	0.53 (0.41, 0.62)
	***P*** **= 0.0001**	***P*** **= 0.0001**	***P*** **= 0.0001**	*P* = 0.06	*P* = 0.7
Day 1: Controls n = 60; SIRS n = 9; sepsis n = 5; severe sepsis n = 28; septic shock n = 74					
**Day 3**					
≥ Sepsis (n = 81)	**0.84 (0.72, 0.96)**	**0.81 (0.70, 0.92)**	0.69 (0.52, 0.87)	0.69 (0.54, 0.83)	0.73 (0.60, 0.87)
	***P*** **= 0.0001**	***P*** **= 0.001**	*P* = 0.03	*P* = 0.04	*P =* 0.009
≥ Severe sepsis (n = 67)	**0.80 (0.70, 0.91)**	0.71 (0.60, 0.81)	0.66 (0.52, 0.80)	0.61 (0.49, 0.74)	0.59 (0.47, 0.72)
	***P*** **= 0.0001**	*P* = 0.003	*P* = 0.02	*P* = 0.1	*P* = 0.2
Septic shock (n = 39)	0.72 (0.61, 0.82)	**0.76 (0.66, 0.87)**	0.66 (0.55, 0.77)	0.72 (0.62, 0.83)	0.57 (0.45, 0.70)
	*P* = 0.0001	***P*** **= 0.0001**	*P* = 0.01	*P* = 0.0001	*P* = 0.3
Day 3: SIRS n = 15; sepsis n = 14; severe sepsis n = 28; septic shock n = 39					
**Day 8**					
≥ Sepsis (n = 48)	**0.82 (0.71, 0.93)**	0.74 (0.61, 0.87)	0.64 (0.50, 0.78)	0.69 (0.54, 0.84)	**0.75 (0.64, 0.87)**
	***P*** **= 0.0001**	*P* = 0.001	*P* = 0.06	*P* = 0.01	***P*** **= 0.001**
≥ Severe sepsis (n = 37)	**0.77 (0.65, 0.88)**	0.73 (0.61, 0.85)	0.68 (0.55, 0.81)	0.65 (0.51, 0.78)	0.71 (0.58, 0.83)
	***P*** **= 0.0001**	*P* = 0.001	*P* = 0.01	*P* = 0.04	*P* = 0.004
Septic shock (n = 18)	**0.79 (0.66, 0.92)**	0.69 (0.55, 0.83)	**0.78 (0.65, 0.92)**	0.67 (0.53, 0.81)	0.68 (0.53, 0.84)
	***P*** **= 0.0001**	*P* = 0.02	***P*** **= 0.001**	*P* = 0.04	*P* = 0.02
Day 8: SIRS n = 28; sepsis n = 11; severe sepsis n = 19; septic shock n = 18					

A minimal area under the curve was set at ≥0.75 (highlighted in bold type). SIRS, systemic inflammatory response syndrome.

Accordingly, presepsin revealed valuable diagnostic goodness criteria at defined cutoff levels (≥sepsis = 530 pg/ml; ≥ severe sepsis = 600 pg/ml; ≥ septic shock = 700 pg/ml) with a minimum sensitivity of 89% to diagnose either patients with at least sepsis, severe sepsis or septic shock at all points in time (Table 4). At day 1, patients with presepsin levels ≥700 pg/ml were up to nine times more likely (that is, relative risk) to suffer from septic shock than patients with lower levels. Specificity was 77%, PPV 74% and the NPV was 92%, thereby reaching a diagnostic test accuracy of 82% (Table 4). At day 3, PPV’s for presepsin levels were at least 82% corresponding to a low number of patients diagnosed false positive. In contrast, at day 8 NPVs for presepsin levels were at least 81%, corresponding to a low number of patients diagnosed false negative (Table 4).

**Table 4:** **Goodness criteria of presepsin for diagnosis of sepsis, severe sepsis and septic shock during the first week of ICU treatment**

	**Area under the curve**	**Cutoff, pg/ml**	**Accuracy, %**	**Sensitivity, %**	**Specificity, %**	**PPV, %**	**NPV, %**	**Relative risk**	***P*** **-value**
**Day 1**									
≥ Septic shock	0.80	700	82	91 (67/74)	77 (78/102)	74 (67/91)	92 (78/85)	8.9	0.0001
**Day 3**									
≥ Sepsis	0.84	530	86	90 (73/81)	60 (09/15)	93 (73/79)	56 (09/16)	2.1	0.0001
≥ Severe sepsis	0.80	600	80	91 (61/67)	54 (15/28)	82 (61/74)	71 (15/21)	2.9	0.0001
**Day 8**									
≥ Sepsis	0.82	530	76	94 (45/48)	46 (13/28)	75 (45/60)	81 (13/16)	4.0	0.001
≥ Severe sepsis	0.77	600	66	92 (34/37)	41 (16/39)	60 (34/57)	84 (16/19)	3.8	0.001
≥ Septic shock	0.79	700	50	89 (16/18)	38 (22/58)	31 (16/52)	92 (22/24)	3.7	0.03

Diagnostic goodness criteria have only been calculated when the diagnostic AUC was ≥0.75. PPV and NPV: positive and negative predictive values. Values in brackets represent the following: sensitivity (true positives/all diseased); specificity (true negatives / all non diseased); PPV (true positives/all test positives); NPV (true negatives/all test negatives).

### Prognostic value of presepsin

All-cause mortality rates were 50% after 30 days (58/116) and 62% after 6 months (72/116). Six months of follow up were completed in all patients. Presepsin levels were significantly higher in patients who died by 30 days or 6 months compared to those who survived (*P* = 0.008).

The prognostic AUCs of presepsin were statistically significant at all time points and for all-cause mortality at 30 days and at 6 months (Table 5). AUCs of presepsin were numerically greater compared to the AUCs of IL-6 (with the exception of day 1 for prediction of 30-day mortality, presepsin AUC = 0.64), however AUCs for presepsin and IL-6 did not differ significantly (*P* >0.05) (Table 5). APACHE II and SOFA scores were the only further significant prognostic indicators of 30-day and 6-month all-cause mortality, whereas these AUCs did not differ significantly from those of presepsin (*P* >0.05).

**Table 5:** **Prognostic performance of biomarkers and ICU scores for 30-day and 6-month all-cause mortality during the first week of ICU treatment**

	**Presepsin**	**Interleukin-6**	**Procalcitonin**	**C-reactive protein**	**Leukocytes**	**SOFA**	**APACHE II**
	**AUC (95% CI)**						
**30-day all-cause mortality**							
**Day 1**	0.64	0.69	0.59	0.54	0.51	0.64	0.70
	(0.54, 0.75)	(0.55, 0.76)	(0.48, 0.69)	(0.43, 0.65)	(0.41, 0.62)	(0.52, 0.77)	(0.60, 0.80)
	***P*** **= 0.008**	***P*** **= 0.001**	*P* = 0.1	*P* = 0.5	*P* = 0.8	***P*** **= 0.05**	***P*** **= 0.0001**
**Day 3**	0.70	0.69	0.58	0.64	0.55	0.65	0.64
	(0.59, 0.81)	(0.58, 0.80)	(0.46, 0.70)	(0.52, 0.76)	(0.42, 0.67)	(0.53, 0.77)	(0.51, 0.77)
	***P*** **= 0.002**	***P*** **= 0.002**	*P* = 0.2	*P* = 0.03	*P* = 0.5	***P*** **= 0.04**	***P*** **= 0.04**
**Day 8**	0.69	0.67	0.57	0.61	0.60	0.69	0.60
	(0.56, 0.82)	(0.54, 0.81)	(0.41, 0.73)	(0.46, 0.75)	(0.46, 0.75)	(0.57, 0.81)	(0.42, 0.78)
	***P*** **= 0.02**	***P*** **= 0.03**	*P* = 0.4	*P* = 0.2	*P* = 0.2	***P*** **= 0.007**	*P* = 0.3
58 of 116 patients died after 30 days corresponding to a 50% all-cause mortality rate.							
**6 months all-cause mortality**							
**Day 1**	0.68	0.66	0.59	0.57	0.54	0.67	0.65
	(0.58, 0.78)	(0.56, 0.76)	(0.49, 0.70)	(0.45, 0.69)	(0.43, 0.65)	(0.55, 0.78)	(0.54, 0.76)
	***P*** **= 0.001**	*P* = 0.05	*P* = 0.1	*P* = 0.2	*P* = 0.5	***P*** **= 0.01**	***P*** **= 0.01**
**Day 3**	0.70	0.70	0.57	0.61	0.59	0.64	0.61
	(0.59, 0.80)	(0.59, 0.80)	(0.45, 0.69)	(0.49, 0.73)	(0.47, 0.71)	(0.52, 0.76)	(0.48, 0.74)
	***P*** **= 0.002**	***P*** **= 0.001**	*P* = 0.3	*P* = 0.08	*P* = 0.2	***P*** **= 0.04**	*P* = 0.1
**Day 8**	0.71	0.67	0.56	0.63	0.60	0.71	0.64
	(0.58, 0.83)	(0.54, 0.80)	(0.42, 0.70)	(0.49, 0.77)	(0.46, 0.74)	(0.60, 0.83)	(0.44, 0.84)
	***P*** **= 0.004**	***P*** **= 0.02**	*P* = 0.4	*P* = 0.06	*P* = 0.2	***P*** **= 0.001**	*P* = 0.2
72 of 116 patients died after 6 months corresponding to a 62% all-cause mortality rate.							

Significant *P*-values (*P* <0.05) are highlighted in bold type. AUC, area under the curve; APACHE II, acute physiology and chronic health evaluation II; SOFA, sequential organ failure assessment.

**Figure 2:**
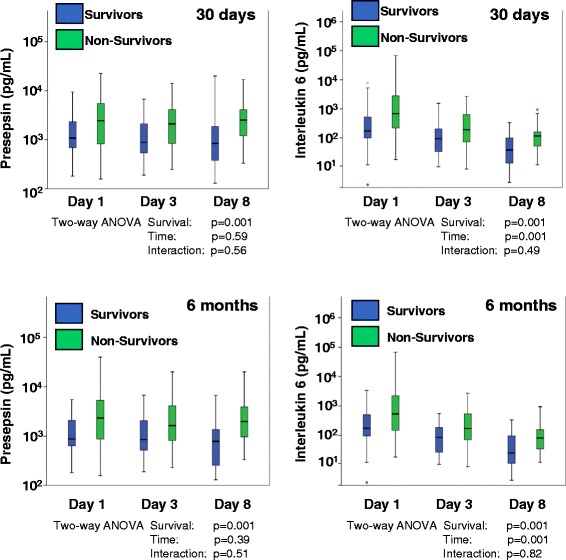
**Presepsin (left column) and IL-6 levels (right column) were significantly increased (** ***P*** **<0.001) in non-survivors compared to survivors after 30 days (top) and 6 months (bottom) consistently at days 1, 3 and 8 of ICU treatment.** IL-6 levels decreased both in survivors and non-survivors during the first week of ICU treatment (survivors: after 30 days, n = 58; 6 months, n = 44; non-survivors: after 30 days, n = 58; 6 months, n = 72; *P* = 0.001). A significant decrease of presepsin levels over days 1, 3 and 8 of ICU treatment was not detected (*P* = 0.59 and *P* = 0.39). Log-transformed biomarker concentrations were analyzed by two-way analysis of variance (ANOVA) to estimate the effects of the two factors, time and survival on biomarker levels. An interaction of survival with time was not detected. Data are presented as medians with 25th and 75th percentiles (boxes), smallest and largest values without extreme values (whiskers) and extreme values (points).

Presepsin and IL-6 levels were significantly (*P* <0.001) increased in non-survivors (30 days, n = 58; 6 months, n = 72) compared to survivors (30 days, n = 58; 6 months, n = 44) consistently at days 1, 3 and 8 of ICU treatment (Figure 2). Presepsin levels of both survivors and non-survivors did not change during the first week of ICU treatment, whereas IL-6 levels decreased (*P* <0.001) both in survivors and non-survivors (Figure 2). An interaction of survival with time was not detected. Specifically, presepsin levels were significantly higher (*P* = 0.02) in patients who died from septic shock within 6 months (presepsin median: 2,690 pg/ml, IQR 1,348 to 6,719 pg/ml, n = 53) compared to those patients surviving septic shock (presepsin median: 1,234 pg/ml, IQR 717 to 3,420 pg/ml, n = 19), when measured at day 1 of ICU treatment (data not shown).

**Figure 3:**
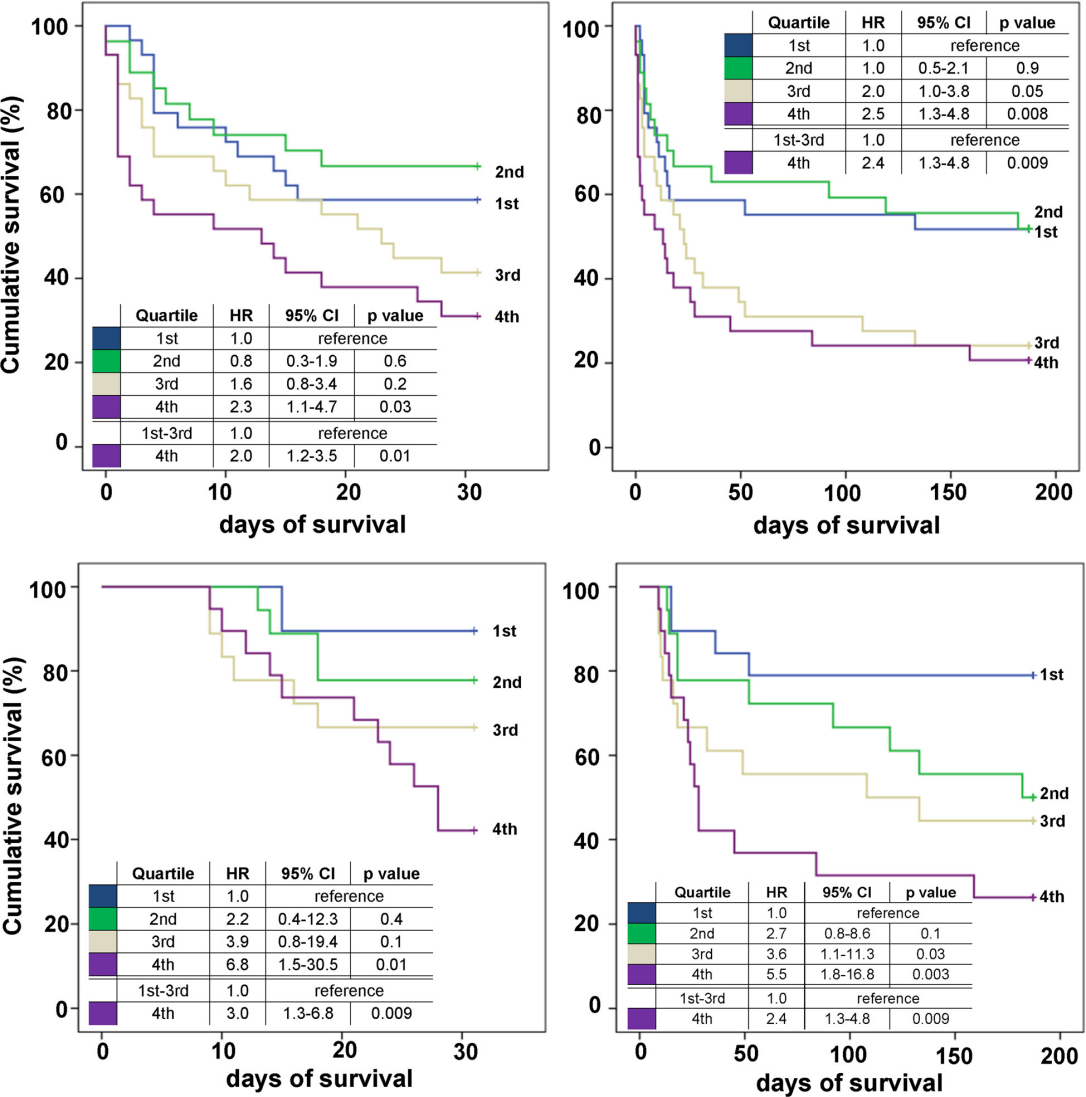
**Kaplan-Meier survival curves evaluated by quartiles of presepsin after 30 days (left column) and 6 months (right column) of follow up in the total study cohort (n = 116).** Hazard ratios (HR) were calculated for each risk group according to presepsin quartiles measured at day 1 (top) and day 8 (bottom).

Patients with the highest presepsin levels in the fourth quartiles were up to 6.8 times more likely to die by 30 days, and up to 5.5 times more likely to die by 6 months both when measured at day 1 or day 8 of intensive care treatment (Figure 3). After adjusting for age, sex, creatinine, ICU days and APACHE II score (model 2), presepsin levels measured at days 1 and 8 revealed significant prognostic value for 30-day and 6-month all-cause mortality (range of HR 1.8 to 7.5; *P* <0.03) (Table 6). Presepsin levels at day 3 were significantly associated with an adverse outcome only in univariate Cox models. Notably, IL-6 offered significant prognostic value for both short- and long-term all-cause mortality at all time points during ICU treatment (Table 6).

**Table 6:** **Cox regression models to predict 30-day and 6-month all-cause mortality at days 1, 3 and 8 of intensive care treatment**

	**Model 1**				**Model 2**			
	**Unadjusted**				**Adjusted for age, sex, creatinine, ICU days, APACHE II**			
**Predictor**	**HR**	**95% CI**	**Chi-square**	***P*** **-value**	**HR**	**95% CI**	**Chi-square**	***P*** **-value**
**All-cause mortality 30 days**								
**Day 1**								
Log presepsin, pg/ml	1.9	1.2, 3.0	6.9	0.009	2.2	1.2, 4.1	42.9	0.02
Log interleukin 6, pg/ml	1.7	1.4, 2.2	20.5	0.0001	1.9	1.4, 2.7	54.7	0.0001
**Day 3**								
Log presepsin, pg/ml	1.8	1.0, 3.3	3.7	0.05	1.3	0.5, 3.3	4.9	0.7
Log interleukin 6, pg/ml	2.6	1.6, 4.4	13.2	0.0001	3.2	2.0, 5.4	28.3	0.0001
**Day 8**								
Log presepsin, pg/ml	2.5	1.3, 5.0	7.4	0.007	7.5	2.4, 23.3	22.6	0.001
Log interleukin 6, pg/ml	3.9	1.6, 9.4	9.2	0.003	6.0	1.9, 19.2	18.3	0.002
**All-cause mortality 6 months**								
**Day 1**								
Log presepsin, pg/ml	2.0	1.3, 3.0	10.6	0.001	2.7	1.5, 4.9	32.0	0.001
Log interleukin 6, pg/ml	1.7	1.3, 2.1	19.3	0.0001	1.7	1.3, 2.3	36.5	0.0001
**Day 3**								
Log presepsin, pg/ml	1.9	1.2, 3.2	6.5	0.01	1.5	0.6, 3.4	3.3	0.4
Log interleukin 6, pg/ml	2.7	1.7, 4.3	16.6	0.0001	3.1	1.9, 4.9	22.3	0.0001
**Day 8**								
Log presepsin, pg/ml	2.6	1.5, 4.3	12.7	0.0001	4.5	2.0, 10.4	17.2	0.0001
Log interleukin 6, pg/ml	3.1	1.6, 6.1	11.5	0.001	3.5	1.5, 7.8	12.3	0.003

Hazard ratios (HR) describe the HR for a biomarker change per log unit-increase. APACHE II, acute physiology and chronic health evaluation II score.

Despite replacing the APACHE II with the SOFA score within Cox regression models to predict 30-day and 6-month all-cause mortality, presepsin retained its significant prognostic value at days 1 and 8 of ICU treatment (range of HR 1.9 to 11.7; at least *P* = 0.04). However, presepsin was not statistically significant in adjusted Cox models at day 3 of ICU treatment. In contrast, IL-6 had significant prognostic value at all time points of ICU treatment and for both 30-day and 6-month all-cause mortality (range of HR 1.8 to 8.5; *P* = 0.0001) (data not shown).

## Discussion

The present study comparatively evaluated the diagnostic as well as short- and long-term prognostic value of sCD14-ST - that is - presepsin, with PCT, IL-6, CRP and WBC in patients with severe sepsis and septic shock during the first week of intensive care treatment at days 1, 3 and 8. To the best of our knowledge, the present results are the first that are based on a stepwise and detailed statistical approach with respect to the diagnostic and prognostic capacity for each inflammatory biomarker, stage of sepsis severity and different time points during ICU treatment.

First, it was demonstrated that measurements of presepsin levels had valuable and consistent diagnostic capacity for the different stages of sepsis severity compared to PCT, IL-6, CRP and WBC during the first week of ICU treatment. Diagnostic cutoffs were set at ≥530 pg/ml for ≥ sepsis, at ≥600 pg/ml for ≥ severe sepsis and ≥700 pg/ml for septic shock reaching at least 89% sensitivity based on a minimum AUC of at least 0.77. IL-6 only had discriminative AUCs (that is, AUC >0.75) for diagnosis of septic shock on day 1 and 3 and for sepsis at day 3. PCT, CRP and WBC mostly failed to have any discriminative capacity for the different groups of sepsis severity at the different time points (AUCs <0.75).

In accordance with the presented results, Liu *et al*. previously demonstrated that presepsin levels had the best diagnostic capacity for diagnosis of sepsis (AUC = 0.82), severe sepsis (AUC = 0.84) and septic shock (AUC = 0.79) compared to PCT (that is, severe sepsis AUC = 0.74; septic shock AUC = 0.78) in 859 patients presenting at the emergency department in Bejing, China [13], while cutoff levels (that is, severe sepsis: presepsin cutoff = 449 pg/ml; septic shock: presepsin cutoff = 550 pg/ml) with lower diagnostic sensitivities (that is, severe sepsis 82%, septic shock 86%) were calculated. Ulla *et al*. [14] evaluated 106 patients presenting at the emergency department with SIRS, sepsis, severe sepsis or septic shock, thereby revealing a lower diagnostic value of presepsin for diagnosis of at least sepsis compared to PCT (that is, diagnosis of ≥ sepsis: presepsin, AUC = 0.70; PCT, AUC = 0.88), while the best diagnostic cutoff of presepsin was 600 pg/ml accompanied by a lower sensitivity of 79%.

In the present study we tried to avoid this inconsistency by choosing optimal and uniform cutoff levels (sepsis 530 pg/ml, severe sepsis 600 pg/ml, septic shock 700 pg/ml) at a maximum achievable sensitivity on days 1, 3 and 8 (that is, at least 89%). This approach was based on clinical considerations to capture as many patients as possible who were truly diseased. Interestingly, within the present analysis presepsin levels had weak diagnostic value for the differentiation of septic shock at day 3 of ICU treatment. This might be explained by a longer half-life of presepsin keeping higher concentrations at least until day 3 of intensive care treatment, which might also being influenced by the presence of acute kidney injury (AKI) in patients with severe sepsis/septic shock [12]. At day 8 of ICU treatment, presepsin again revealed diagnostic discrimination for patients with septic shock.

In contrast, Endo *et al*. [11,21] evaluated presepsin, IL-6 and PCT levels in patients with proven bacterial infections compared to patients with non-bacterial infections and found that all inflammatory biomarkers were significantly higher in patients with bacterial infections. Corresponding diagnostic AUCs for a proven bacterial infection were highest for presepsin and PCT (that is, presepsin, AUC = 0.908; PCT, AUC = 0.905) and lowest for IL-6 (that is, IL-6, AUC = 0.825).

Recent trials mostly evaluated single measurements of presepsin in patients presenting to the emergency department, trying to establish presepsin as an early one-shot guiding biomarker for emergency care [13,14]. However, too little is yet known about presepsin measurements during the course of severe sepsis or septic shock after immediate admission to the ICU and following intensive care treatment [22]. Accordingly, stepwise diagnostic and prognostic evaluation of the individual patient with severe sepsis is recommended as early as possible during ICU treatment in order to establish realistic treatment goals and clinical decision-making [1]. The present study showed that presepsin levels have several significant associations with different ICU parameters reflecting the intensity and severity of severe sepsis/septic shock within our cohort (for example, significant correlations with APACHE II and SOFA score, days of intensive care treatment, days of mechanical ventilation and days of catecholamine treatment).

Accordingly, further influencing factors on presepsin despite age and renal function are rarely known [12]. Notably, a significant correlation between presepsin and bilirubin, as shown in the present study, has not been described yet. While the liver represents a central organ of metabolic and immunological homeostasis, liver dysfunction can facilitate the progression of multiple organ failure in septic patients [3,23]. Extra-hepatic bacterial infections within sepsis have been described to account for almost 20% of jaundice cases, and increases of bilirubin themselves have been found recurrently in patients with sepsis [3,23]. A correlation between presepsin and inflammatory biomarkers, such as CRP, WBC, IL-6 and PCT, can be explained by the ongoing systemic activation of inflammatory biomarkers during severe sepsis and septic shock [3,24].

Second, it was demonstrated that measurements of presepsin levels revealed valuable prognostic capacity to predict short- and long-term (that is, 30-day and 6-month) all-cause mortality compared to PCT, CRP and WBC consistently throughout days 1, 3 and 8 of ICU treatment. Patients with presepsin levels of the fourth quartile were up to five to seven times more likely to die after 6 months than patients with lower levels. APACHE II and SOFA scores solely revealed acceptable prognostic values for all-cause mortality. IL-6 was the only inflammatory biomarker with comparable prognostic value at all time points as demonstrated both in *C*-statistics and within multivariable Cox regression models being adjusted to age, sex, intensive care days and APACHE II/SOFA score.

The prognostic value of presepsin in severe sepsis/septic shock has not yet been evaluated in detail. Presepsin has been described as a powerful prognostic biomarker compared to PCT and APACHE II scores for short-term 28-day all-cause mortality (presepsin, AUC = 0.66; PCT, AUC = 0.68, APACHE II, AUC = 0.72) [11,13]. Ulla *et al*. [14] demonstrated increased risk of death within 60 days in patients with increased presepsin levels ≥1,000 pg/ml [13,14]. In a retrospective analysis including patients with severe sepsis and septic shock after discharge from the ICU, Masson *et al*. demonstrated constantly increased presepsin levels in decedents and revealed significant prognostic value for both 28-day and 90-day all-cause mortality, whereas PCT failed to provide any prognostic information [22]. It was speculated that decreasing presepsin levels in surviving patients might indicate a beneficial effect of ICU treatment. However, these studies did not include IL-6 measurements as another prognostic biomarker next to presepsin.

Therefore, the present study delivered new evidence about both presepsin and IL-6 as powerful prognostic biomarkers of short- and long-term prognosis in patients with severe sepsis and septic shock [25-27]. Specifically long-term prognostic risk stratification has become of major interest for patients with severe sepsis and septic shock [14,22,28]. Patients surviving severe sepsis/septic shock remain at increased risk of death in the following months and years after hospital discharge, while suffering from chronically impaired physical or neurocognitive functioning [3]. In this respect, measurements of presepsin and IL-6 during the first week of ICU treatment might help to better prognosticate the individual half-year course of these patients, specifically addressing the risk of all-cause mortality.

### Limitations

The present study was performed on the internal ICU at the University Medical Centre Mannheim, Germany. Primarily, this study included patients with severe sepsis or septic shock 24 h after admission to the medical ICU or 24 h after disease onset during ICU treatment. If statistically significant diagnostic and prognostic values for presepsin and IL-6 were calculated, sufficient statistical power of at least 80% could have been presumed. If statistically non-significant differences were calculated, a larger number of study patients of at least 300 to 2,000 patients in each group would have been required to guarantee sufficient statistical power, for example, for the inflammatory biomarkers PCT, CRP and WBC with respect to diagnosis or prognosis. However, high *P*-values and relatively marginal differences in these biomarker levels with regard to diagnostic and prognostic differentiation in the present study cohort of 116 patients suggest that there might not be a real clinically relevant difference. Within our study, we did not find any significant associations of presepsin levels with age and sex of the patients, which were previously described [12]. Analyses were not blinded with respect to WBC and CRP, because both biomarkers are used in daily routine practice. Based on the presented results, a single or double biomarker combination (for example, presepsin and IL-6) for diagnostic and prognostic assessment appears to be useful in patients with severe sepsis or septic shock being treated on a medical ICU. Additional clinical tools, such as risk-prediction scores (for example, APACHE II or SOFA score) might add specific information when single non-significant or negative biomarker results (for example, CRP, WBC, PCT) are evident. However, combined biomarker strategies for diagnostic and prognostic assessment need to be investigated, both in larger, multi-center studies and in prospective clinical studies, with internal medicine patients as well as surgical or trauma patients.

## Conclusions

Taken together, it has been demonstrated that measurements of presepsin levels had independent diagnostic and prognostic value in patients with severe sepsis and septic shock during the first week of intensive care treatment. Presepsin levels had valuable diagnostic value for the diagnosis of sepsis, severe sepsis and septic shock at days 1, 3 and 8 of ICU treatment compared to PCT, IL-6, CRP and WBC. Additionally, presepsin levels had valuable prognostic capacity to predict short- and long-term all-cause mortality when compared to PCT, IL-6, CRP, WBC and APACHE II score.

## Key messages

- Presepsin reveals valuable diagnostic capacity for stages of sepsis severity compared to PCT, IL-6, CRP, and WBC in patients being treated on an internal ICU.
- Diagnostic cutoffs of presepsin were set at ≥530 pg/ml for ≥ sepsis, at ≥600 pg/ml for ≥ severe sepsis and ≥700 pg/ml for septic shock.
- Presepsin levels had valuable prognostic capacity to predict short- and long-term all-cause mortality at 30 days and 6 months compared to PCT, CRP, WBC, SOFA and APACHE II scores.
- IL-6 had comparable prognostic value to presepsin levels.
- Diagnostic and prognostic capacity of presepsin was consistently demonstrated throughout days 1, 3 and 8 of ICU treatment.

## Abbreviations

ANOVA: analysis of variance
APACHE II: acute physiology and chronic health evaluation II
AUC: area under the curve
CD: cluster of differentiation
CRP: C-reactive protein
EDTA: ethylenediaminetetraacetic acid
FiO_2_: fraction of inspired oxygen
HR: hazard ratio
IL-6: interleukin 6
PaO_2_: partial pressure of oxygen
PCT: procalcitonin
ROC: receiver-operating characteristic
sCD14-ST: soluble cluster of differentiation 14 subtype
SEM: standard error of mean
SIRS: systemic inflammatory response syndrome
WBC: white blood cells

## References

[1] Dellinger RP, Levy MM, Rhodes A, Annane D, Gerlach H, Opal SM, Sevransky JE, Sprung CL, Douglas IS, Jaeschke R, Osborn TM, Nunnally ME, Townsend SR, Reinhart K, Kleinpell RM, Angus DC, Deutschman CS, Machado FR, Rubenfeld GD, Webb SA, Beale RJ, Vincent JL, Moreno R (2013). Surviving sepsis campaign: international guidelines for management of severe sepsis and septic shock: 2012. Crit Care Med.

[2] Adhikari NK, Fowler RA, Bhagwanjee S, Rubenfeld GD (2010). Critical care and the global burden of critical illness in adults. Lancet.

[3] Angus DC, van der Poll T (2013). Severe sepsis and septic shock. N Engl J Med.

[4] Kibe S, Adams K, Barlow G (2011). Diagnostic and prognostic biomarkers of sepsis in critical care. J Antimicrob Chemother.

[5] Sridharan P, Chamberlain RS (2013). The Efficacy of Procalcitonin as a Biomarker in the Management of Sepsis: Slaying Dragons or Tilting at Windmills?. Surg Infect (Larchmt).

[6] Wacker C, Prkno A, Brunkhorst FM, Schlattmann P (2013). Procalcitonin as a diagnostic marker for sepsis: a systematic review and meta-analysis. Lancet Infect Dis.

[7] Shozushima T, Takahashi G, Matsumoto N, Kojika M, Okamura Y, Endo S (2011). Usefulness of presepsin (sCD14-ST) measurements as a marker for the diagnosis and severity of sepsis that satisfied diagnostic criteria of systemic inflammatory response syndrome. J Infect Chemother.

[8] Okamura Y, Yokoi H (2011). Development of a point-of-care assay system for measurement of presepsin (sCD14-ST). Clin Chim Acta.

[9] Shirakawa K, Naitou K, Hirose J, Takahashi T, Furusako S (2011). Presepsin (sCD14-ST): development and evaluation of one-step ELISA with a new standard that is similar to the form of presepsin in septic patients. Clin Chem Lab Med.

[10] Sugie Y, Igami K, Shoji K, Arai N, Tazaki Y, Kouta H, Okamura Y, Tashiro S, Yokoi H (2011). Performance evaluation of the new rapid fertility assays in whole blood and plasma on PATHFAST. Clin Lab.

[11] Endo S, Suzuki Y, Takahashi G, Shozushima T, Ishikura H, Murai A, Nishida T, Irie Y, Miura M, Iguchi H, Fukui Y, Tanaka K, Nojima T, Okamura Y (2012). Usefulness of presepsin in the diagnosis of sepsis in a multicenter prospective study. J Infect Chemother.

[12] Chenevier-Gobeaux C, Trabattoni E, Roelens M, Borderie D, Claessens YE (2013). Presepsin (sCD14-ST) in emergency department: The need for adapted threshold values?. Clin Chim Acta.

[13] Liu B, Chen YX, Yin Q, Zhao YZ, Li CS (2013). Diagnostic value and prognostic evaluation of Presepsin for sepsis in an emergency department. Crit Care.

[14] Ulla M, Pizzolato E, Lucchiari M, Loiacono M, Soardo F, Forno D, Morello F, Lupia E, Moiraghi C, Mengozzi G, Battista S (2013). Diagnostic and prognostic value of presepsin in the management of sepsis in the emergency department: a multicenter prospective study. Crit Care.

[15] **American College of Chest Physicians/Society of Critical Care Medicine Consensus Conference: definitions for sepsis and organ failure and guidelines for the use of innovative therapies in sepsis.** *Crit Care Med* 1992, **20:**864–874. 1597042

[16] Reinhart K, Brunkhorst FM, Bone HG, Bardutzky J, Dempfle CE, Forst H, Gastmeier P, Gerlach H, Grundling M, John S, Kern W, Kreymann G, Kruger W, Kujath P, Marggraf G, Martin J, Mayer K, Meier-Hellmann A, Oppert M, Putensen C, Quintel M, Ragaller M, Rossaint R, Seifert H, Spies C, Stuber F, Weiler N, Weimann A, Werdan K, Welte T (2010). German Interdisciplinary Association for I, Emergency Care M and German Sepsis S [Prevention, diagnosis, treatment, and follow-up care of sepsis. First revision of the S2k Guidelines of the German Sepsis Society (DSG) and the German Interdisciplinary Association for Intensive and Emergency Care Medicine (DIVI)]. Anaesthesist.

[17] Knaus WA, Draper EA, Wagner DP, Zimmerman JE (1985). APACHE II: a severity of disease classification system. Crit Care Med.

[18] Vincent JL, Moreno R, Takala J, Willatts S, De Mendonça A, Bruining H, Reinhart CK, Suter PM, Thijs LG (1996). The SOFA (Sepsis-related Organ Failure Assessment) score to describe organ dysfunction/failure. On behalf of the Working Group on Sepsis-Related Problems of the European Society of Intensive Care Medicine. Intensive Care Med.

[19] Kurihara T, Yanagida A, Yokoi H, Koyata A, Matsuya T, Ogawa J, Okamura Y, Miyamoto D (2008). Evaluation of cardiac assays on a benchtop chemiluminescent enzyme immunoassay analyzer, PATHFAST. Anal Biochem.

[20] Hanley JA, McNeil BJ (1983). A method of comparing the areas under receiver operating characteristic curves derived from the same cases. Radiology.

[21] Vodnik T, Kaljevic G, Tadic T, Majkic-Singh N (2013). Presepsin (sCD14-ST) in preoperative diagnosis of abdominal sepsis. Clin Chem Lab Med.

[22] Masson S, Caironi P, Spanuth E, Thomae R, Panigada M, Sangiorgi G, Fumagalli R, Mauri T, Isgro S, Fanizza C, Romero M, Tognoni G, Latini R, Gattinoni L (2014). Presepsin (soluble CD14 subtype) and procalcitonin levels for mortality prediction in sepsis: data from the Albumin Italian Outcome Sepsis trial. Crit Care.

[23] Bauer M, Press AT, Trauner M (2013). The liver in sepsis: patterns of response and injury. Curr Opin Crit Care.

[24] Henriquez-Camacho C, Losa J (2014). Biomarkers for Sepsis. BioMed Res Int.

[25] Bloos F, Reinhart K (2014). Rapid diagnosis of sepsis. Virulence.

[26] Harbarth S, Holeckova K, Froidevaux C, Pittet D, Ricou B, Grau GE, Vadas L, Pugin J (2001). Diagnostic value of procalcitonin, interleukin-6, and interleukin-8 in critically ill patients admitted with suspected sepsis. Am J Respir Crit Care Med.

[27] Sakr Y, Burgett U, Nacul FE, Reinhart K, Brunkhorst F (2008). Lipopolysaccharide binding protein in a surgical intensive care unit: a marker of sepsis?. Crit Care Med.

[28] Huang DT, Angus DC, Barnato A, Gunn SR, Kellum JA, Stapleton DK, Weissfeld LA, Yealy DM, Peake SL, Delaney A, Bellomo R, Cameron P, Higgins A, Holdgate A, Howe B, Webb SA, Williams P, Osborn TM, Mouncey PR, Harrison DA, Harvey SE, Rowan KM (2013). Harmonizing international trials of early goal-directed resuscitation for severe sepsis and septic shock: methodology of ProCESS, ARISE, and ProMISe. Intensive Care Med.

